# Molecular Mechanisms of Nasal Epithelium in Rhinitis and Rhinosinusitis

**DOI:** 10.1007/s11882-014-0495-8

**Published:** 2014-12-11

**Authors:** Sanna Toppila-Salmi, Cornelis M. van Drunen, Wytske J. Fokkens, Korneliuz Golebski, Pirkko Mattila, Sakari Joenvaara, Jutta Renkonen, Risto Renkonen

**Affiliations:** 1 Haartman Institute, University of Helsinki, Haartmaninkatu 3, P.O. Box 21, 00014 Helsinki, Finland; 2 Department of Allergy, Helsinki University Central Hospital, Helsinki, Finland; 3 Department of Otorhinolaryngology, Academic Medical Center, Meibergdreef, 1105 AZ Amsterdam, The Netherlands

**Keywords:** Airway, Allergic rhinitis, Allergy, Epithelium, Nasal polyp, Respiratory, Rhinitis, Rhinosinusitis, Sinusitis

## Abstract

Allergic rhinitis, nonallergic rhinitis, and chronic rhinosinusitis are multifactorial upper airway diseases with high prevalence. Several genetic and environmental factors are proposed to predispose to the pathogenesis of the inflammatory upper airway diseases. Still, the molecular mechanisms leading toward the onset and progression of upper airway diseases are largely unknown. The upper airway epithelium has an important role in sensing the environment and regulating the inhaled air. As such, it links environmental insults to the host immunity. Human sinonasal epithelium serves as an excellent target for observing induced early-phase events, in vivo, and with a systems biological perspective. Actually, increasing number of investigations have provided evidence that altered homeostasis in the sinonasal epithelium might be important in the chronic upper airway inflammation.

## Introduction

Allergic rhinitis (AR), nonallergic rhinitis (NAR), and chronic rhinosinusitis (CRS) are multifactorial chronic inflammatory upper airway diseases with high prevalence and large variation in their characters. The diseases are affected by each other, by lower airway diseases, by the composition of inhaled air, infections, the sensitization profile, and many genetic and lifestyle-related factors [1–6]. Allergic rhinitis (AR) is an IgE-mediated symptomatic inflammation with a prevalence of 10–20 % in worldwide population [1]. In tropical urban environment, the major cause for AR is mite monosensitization, whereas in other climate zones, multiple sensitizations to several allergen types predominate [7, 8]. Nonallergic noninfectious rhinitis (NAR) has variable etiology and a prevalence of 5–10 % worldwide [9, 10]. The etiology underlies local allergic rhinitis, hormonal, and medication-induced conditions, among others [9–11]. A subgroup of patients with NAR has fluctuating nasal hyperreactivity induced by many irritants and temperature changes without a defined etiology. Its mechanism might be related to a dysfunction of autonomous nervous system [12]. Chronic rhinosinusitis (CRS) is a chronic symptomatic inflammation of the sinonasal tract, with prevalence around 10 % [13]. CRS presents with or without nasal polyps (NP). Patients with nonsteroidal anti-inflammatory drug (NSAID)-exacerbated respiratory disease (e.g., NERD) tend to have more severe forms of CRS with nasal polyps and asthma.

The pathomechanisms of inflammatory airway diseases are connected to the large biological networks between the environment and the host. During development, host genetics and environmental factors can significantly modulate the barrier homeostasis, thus influencing the predilection toward chronic inflammation of the airways. The respiratory epithelium has important innate immunity functions. It also mediates parts of the innate and adaptive immunity by its antigen presentation, phagocytosis, and pattern recognition abilities. Thus, altered airway barrier functions might have a pivotal role in the pathogenesis of chronic airway inflammation. Human nasal epithelium and secretions are easily accessible. Thus, they provide an excellent target for systems level studies on inflammation [88]. This is possible particularly in patients with seasonal rhinitis because the triggering factors can be identified [14, 15]. It has been suggested that investigations on nasal epithelial interactions might provide additional knowledge for the lower airway inflammation [16]. Of note, nerve endings and immune cells might be fundamental in response to multiple environmental factors [14, 17, 18]. However, in this review, the authors mainly focus on the biology of human upper airway epithelium in AR, NAR, and CRS.

## Genomics of the Upper Airway Diseases with Epithelial Context

There is a clear hereditary component in allergic rhinitis that has been well corroborated by segregation studies and investigations in twins [19, 20]. However, much less evidence exists on inheritage of NAR [21] and of CRS [22]. Genome-wide association studies (GWAS) have reported 22 significant AR-associating loci [23, 24, 25•, 26•, 27]. Based on our unpublished results of RNA-sequencing for nasal brushings, less than half of these loci could have epithelial function. Yet, their role in the epithelium and other cell types during the development of AR has not been resolved. As an example, FOXA1 (25) is connected to cell differentiation. It can increase the expression of mucin 2 gene in goblet cells [28]. TPD52 [25•] is expressed predominantly by epithelial cells and has a function in the development or maintenance of the epithelial cell phenotype, in cell proliferation, migration, and cell death [29]. ID2 [25•] binds TGF-β and takes part in cell differentiation, apoptosis, and epithelial to mesenchymal transition [30]. IL4R [25•] has a role in the regeneration and in enhancing MUC4 in respiratory epithelium. It binds IL4 and IL13, which promote IgE production and differentiation of Th2 cells [31]. ETS1 [25•] is a transcription factor particularly expressed in basal cells of airway epithelium. It is important in stem cell development, cell senescence, and death [32]. TLR1-TLR6-TLR10 [25•] and NOD1 [23] are pattern recognition receptors found in nasal epithelium [33]. Interestingly, Nilsson et al. performed a replication study in children with AR and showed that TLR6-TLR1 locus is likely to have a central role in the development of allergic disease [34]. Still, further studies are needed to show, whether these AR-associating loci are important in epithelial pathology during airway allergy.

Bunyavanich et al. studied further the biologic context for AR-associating loci identified by GWAS, by expression quantitative trait loci (eQTL) and expression single nucleotide polymorphism (eSNP) mapping, as well as by network and pathway analyses [26•]. Interestingly, they recognized enrichment in mitochondrial pathways [26•]. Previous studies have detected the mitochondrial dysfunction of airway epithelium during bacterial and viral infections, cigarette smoking, and asthma [35].

Two pooling-based GWASes are known for the CRS phenotype. A study discovered three loci that consistently associate with CRS, yet their function in sinonasal epithelium is not confirmed: TCF7L2 [36] participates in the Wnt signaling pathway and modulates MYC expression. It maintains the epithelial stem cell compartment of the small intestine. AOAH [36] has been replicated significantly in a Chinese population [37]. It is expressed in epithelium and leukocytes. It detoxifies gram-negative bacteria by hydrolyzing acyloxylacyl-linked fatty acyl chains of lipopolysaccharides. TP73 [38] mediates oxidative stress response, cell differentiation, and remodeling in nasal polyp epithelium [39]. The group of Desrosiers performed also RNA array analysis and detected upregulation of the expression of the LAMB1 gene and the laminin pathway, in differentiated primary epithelial cells from CRS patients, suggesting a role for extracellular matrix genes in the development of CRS [40]. To the knowledge of the authors, there are no GWAS studies on NAR phenotypes.

### The Interplay Between Inhaled Particles and Epithelial Transcriptome

A great variety of allergen-, host-, and environment-dependent mechanisms facilitates allergen and pathogen entry into the respiratory mucosa. Allergens have proteolytic, lipid-binding, and microbial-mimicking properties which enable their entry [41]. Penetration of mite allergens, for instance, associates with aberrant host functions such as pattern recognition, calcium metabolism, and cell-cell contacts [42, 43•, 44, 45]. We have demonstrated by electron microscopy and proteomics that birch pollen allergens (Bet v1) were able to bind plasma membrane lipid rafts and were rapidly transported through the epithelium in caveolar vesicles to meet a mast cell solely in patients allergic to birch [46]. Microarray experiments have shown that pollen exposure causes greatest fold changes in the nasal epithelial transcripts that belong to immunology category in controls, whereas response to virus and cellular transportation are abundant categories in pollen allergic subjects [15, 47].

We performed RNA microarray of cultured epithelial cells from bronchial brushings and nasal biopsies and showed that about 2000 genes were differentially expressed between healthy lower and upper airway epithelium, whereas in allergic rhinitis with or without asthma, this was only 40 and 301 genes, respectively. Genes influenced by allergic rhinitis with or without asthma were linked to lung development, remodeling, regulation of peptidases, and normal epithelial barrier functions [48]. We also stimulated primary nasal and bronchial epithelial cells from the same individuals by a viral double-stranded RNA (dsRNA) analog poly (I:C) and identified gene expression profiles by RNA-array analysis. Asthma patients demonstrated significantly fewer induced genes, exhibiting reduced downregulation of mitochondrial genes. The majority of genes related to viral responses appeared to be similarly induced in upper and lower airways in all groups. However, the induction of several interferon-related genes was impaired in patients with asthma [48]. Interestingly, expression profiling showed resemblance in the cytokine profiles of EGR1, DUSP1, FOSL1, JUN, MYC, and IL6 after stimulation of airway epithelial cells with either dsRNA or with house dust mite; however, both triggers also induced a specific response (e.g., ATF3, FOS, and NFKB1) [49•]. This and other studies suggest that the risk for microbial infection and its underlying immune dysfunction might be a phenotypic or clinical feature of both atopic and nonatopic chronic conditions in the airways than only a secondary effect [5].

### Modulation of Transcriptome in Upper Airway Epithelium

There are several molecular mechanisms by which environmental factors might regulate gene expression. Epigenetic mechanisms alter gene expression without altering the underlying DNA sequence [50]. The most studied epigenetic mechanisms are DNA methylation and histone modifications. Early infections together with other factors may shape developing immunity via epigenetic programming, which is partly inherited [51, 52]. Buro-Auriemma et al. showed that cigarette smoking induces small airway epithelial epigenetic changes with corresponding modulation of gene expression [53]. So far, epithelial epigenetics in upper airways have not been studied on systems level.

Recent genome-wide studies confirm that much of the DNA that does not encode proteins encode various types of functional RNAs, which are important players in gene regulation. MicroRNAs (miRNA) are small noncoding RNAs and important fine tuners of immune systems [54]. By binding to its target transcripts, miRNA is able to decrease translation [54]. Rager performed RT-PCR on nasal epithelial samples from macaques that inhaled formaldehyde and detected significant modification in miRNA expression profiles, which influence apoptosis signaling [55]. McKiernan examined long noncoding RNA transcripts (lncRNAs) from bronchial brushings by microarray analysis and found that 1063 out of over 30,000 lncRNAs had different expression between cystic fibrosis and noncystic fibrosis individuals [56]. Thus, it seems that pathologic processes in the airway epithelium are partly driven by noncoding RNAs, which might alter the regulation of gene expression.

## Disrupted Epithelium During Chronic Upper Airway Diseases

Airway epithelium has important functions that mediate innate immunity actions, maintain cell integrity, and promote self-renewal. These functions seem to be essentially involved in the development of human chronic upper airway disorders.

### Mucociliary Clearance

Cilia are microtubule-based hair-like organelles that play many important roles in development and physiology, and they are implicated in a rapidly expanding spectrum of human diseases, collectively termed ciliopathies. Mucociliary clearance (MCC) is crucial in defense against inhaled insults. Coordinated ciliary beating transports debris-laden mucus from respiratory passages toward the oropharynx [57]. Primary ciliary dyskinesia (PCD) is a congenital ciliopathy with complexity in genetics. Choksi et al. performed whole-transcriptome microarray on zebrafish cilia model. After Foxj1 induction, the corresponding proteins of the modified transcripts are required for ciliary motility and ciliogenesis, such as tubulin-modifying enzymes, components of the transition zone, and intraflagellar transport [58]. Moreover, they performed PANTHER protein characterization and identified cytoskeletal proteins that could serve as structural components of cilia (78 genes), possible regulators of ciliary differentiation and function, including transcriptional regulators (26 genes) and protein regulators such as kinases (19 genes), phosphatases (8 genes), and enzyme modulators (61 genes), suggesting that motile cilia may perform more sensory and signaling functions than previously recognized.

Patients suffering from PCD have typically recurrent and prolonged bacterial infections in the airways, chronic rhinosinusitis with nasal polyps, and chronic alterations in the middle ear and lower airways. It implicates the importance of ciliary function in preventing infections and chronic airway inflammation. Ciliary beating frequency increases in response to multiple host and environmental stimuli through several second messenger pathways including intracellular Ca2+ and NO production [57]. A bitter taste receptor, T2R38, is expressed in human upper respiratory epithelium and is activated in response to acyl-homoserine lactone quorum-sensing molecules secreted by gram-negative bacteria such as *Pseudomonas aeruginosa*. Receptor activation regulates calcium-dependent NO production, resulting in stimulation of mucociliary clearance and direct antibacterial effects. T2R38 taste receptor polymorphism associated with susceptibility to human sinonasal gram-negative bacterial infection [59]. Li et al. have been successful in the culture of human nasal epithelial cells derived from stem cells which are isolated from nasal biopsies in patients with CRSwNP and without PCD diagnosis. They found a significant association of aberrant ciliogenesis with primary motile cilia impairment, that is more frequent in patients with CRSwNP than previously thought [60•]. Prostaglandin E2 (PGE2) has shown to be important for normal mucociliary function in vitro and in animal models [61, 62]. PGE2 synthase knockout mice have prostaglandin E2 deficiency which leads to a phenotype of aspirin sensitivity [63].

### Ion Channels and Mucus

Cilia are coated with membrane-spanning mucins and tethered mucopolysaccharides that exclude mucus from the periciliary space and promote formation of distinct mucus layer [64]. Secretory cells produce a different class of mucins, the polymeric gel-forming mucins. The two major airway gel-forming mucins are MUC5AC and MUC5B. Some secretory cells, known as mucous or goblet cells, produce mucins and store them within easily visualized collections of mucin granules, whereas other cells produce and secrete mucins (especially MUC5B) but lack prominent granules. Gel-forming mucins are secreted into the airway lumen and are responsible for the characteristic viscoelastic properties of the mucus gel layer [65]. Cystic fibrosis (CF) is a lethal multisystem, autosomal recessive disorder. Over 1900 mutations have been identified in the cystic fibrosis transmembrane conductance regulator (CFTR) gene, which encodes an ATP-regulated chloride channel and is present within the apical surface of epithelial cells [56]. The respiratory characters of CF are decreased airway surface liquid volume, increased mucus viscosity, pathogen colonization, impaired protease-antiprotease balance, pulmonary inflammation, and CRSwNP, yet the pathomechanisms are not fully understood [56].

Birket et al. performed microoptical coherence tomography on wild-type and CF piglet trachea and demonstrated a direct link between periciliary liquid (PCL) hydration and mucociliary transport (MCT) rates, a relationship frequently invoked but never experimentally confirmed. However, in CF airways, this relationship was completely disrupted, with greater PCL depths associated with slowest transport rates. This disrupted relationship was recapitulated by selectively inhibiting bicarbonate transport in vitro and ex vivo. CF mucus exhibited increased viscosity in situ due to the absence of bicarbonate transport, explaining defective MCT that occurs even without infection, inflammation, or aberrant PCL hydration [66]. Vasoactive intestinal peptide (VIP) is a neurotransmitter that has been shown to increase the ciliary beat frequency and CF transmembrane conductance regulator-dependent fluid secretion. The detected upregulation of VIP in allergy could contribute to allergic rhinorrhea whereas a loss of VIP-activated secretion in patients with CF may impair mucociliary transport, contributing to increased incidences of sinonasal infections and rhinosinusitis [59]. Apical potassium channels regulate ion transport in airway epithelial cells and influence air surface liquid hydration and mucociliary clearance. Two airway epithelial potassium channels have been discovered as susceptibility loci associating to pediatric CRS [67]. Studies are still needed to evaluate the function of potassium channels in CRS pathogenesis. The allergic rhinitis mucus proteome showed a decrease in antiprotease activity which could affect the epithelial barrier during the insult of allergen proteases [68].

### Antimicrobial Proteins and Immunoglobulin A

Both mucosal and glandular epithelial secrete a large array of molecules that are known to kill or neutralize microorganisms. Disruption of this antimicrobial action might lead to the risk of colonization or infection by microorganisms, which might associate to pathogenesis chronic airway inflammation. Epithelial host defense molecules include small peptides that lyse bacteria and enzymes. Lysozyme targets glycosidic bonds in the peptidoglycan cell wall of bacteria, typically leading to an enzymatic lysis, and is highly effective against many common upper airway gram-positive bacteria, such as streptococci [69]. Lactoferrin sequesters iron, leading to the inhibition of bacterial growth. Secretory leukocyte proteinase inhibitor kills both gram-positive and gram-negative bacteria. Defensins are small cysteine-rich cationic proteins that are directly toxic to many bacteria, fungi, and viruses. They form pore-like structures in microbial membranes leading to efflux of essential ions and nutrients [69]. The S100 proteins comprise a multigene family consisting of more than 20 low-molecular weight proteins that have numerous effects, including influences on cell differentiation and transformation, barrier function, and direct antimicrobial actions. S100A7 kills *Escherichia coli* and is reduced in nasal fluid during allergic rhinitis [69]. S100A7 and S100A8/S100A9 is reduced in both patients with CRSsNP and CRSwNP [69].

SPINK5 is a serine protease inhibitor regulating proteases that might compromise barrier [70]. Highly reduced expression of SPINK has been observed in the epithelial cells taken from patients with CRS. Decrease in the number of glands in nasal polyps of patients with CRSwNP may lead to a localized defect in the production of antimicrobial proteins such as lactoferrin, lysozyme, and proteins of the PLUNC family [71]. Wei et al. performed immunohistochemistry, qRT-PCR, Western blot, and ELISA and demonstrated reduced antimicrobial SPLUNC1 expression and numbers of submucosal glands in the eosinophilic CRSwNP subset compared with the noneosinophilic subset. Moreover, SPLUNC1 expression in polyp epithelial cells was significantly inhibited by IL-4 and IL-13 stimulation in vitro but was significantly upregulated after stimulation with toll-like receptor agonists and glucocorticoids [72]. Epithelial polymeric Ig receptor expression is decreased in patients with CRSwNP and AR, which results in decreased secretory component and IgA antibodies in nasal secretions in parallel to subepithelial accumulation of IgA. This defect in mucosal immunity was associated with eosinophilic, Th2-related inflammation [73].

### Pattern Recognition Receptors

Toll-like receptors (TLR1-TLR10), RIG-like receptors and dectins are among the most known pattern recognition molecules. When they get into contact with the environment, they rapidly detect microbe-associated molecular patterns and either maintain homoeostasis or induce immune responses. Lipopolysaccharide (LPS) of gram-negative bacteria is a molecular pattern recognized by the host through the transmembrane TLR4 and myeloid differentiation antigen (MD-2). Animal models from the lower airways showed that house dust mite allergen, Der p 2, is an MD-2 analog. By binding LPS and epithelial TLR4, Der p 2 may activate pro-inflammatory signaling and stimulate IgE production and allergic reaction [74]. Fransson et al. demonstrated mRNA and protein expression of TLRs 2, 3, and 4 in the nasal mucosa both in subjects with and without birch and/or timothy pollen allergy [75].

Tengroth et al. demonstrated by RTq-PCR immunohistochemistry and flow cytometry that nasal apical epithelium expresses abundantly TLR3, TLR7, TLR9, RIG-I, and MDA-5. Moreover, they showed by ELISA upregulated cytokines (IL-6, GMCSF, IL-8, IFN-b) in the nasal mucosa after stimulation of several TLR agonists, which suggests that epithelial TLR and RLR receptors might mediate nasal viral response and thus could be important in exacerbations [76•]. The group also analyzed by flow cytometry and Luminex naïve nasal polyp and turbinate tissues as well as human tissues after in vivo and in vitro stimulation with a TLR9 agonist, CpG. Interestingly, epithelial expression of TLR9 was detected in turbinates from healthy controls and in polyp tissue, whereas TLR9 was absent in turbinates from CRSwNP patients. CpG stimulation resulted in an upregulation of TLR9 and modulation of cytokines in turbinate tissue from patients, suggesting that defects in the TLR9-mediated microbial defense in the turbinate might explain virus-induced polyp growth [77].

### Signaling and Protein Synthesis

Upper airway epithelium secretes several cytokines including TSLP, IL33, and IL-25. These cytokines are released by tissue damage, pathogen recognition, or allergen exposure. They effect on Th2 cell function either directly or via innate lymphoid cells, which in turn produce IL-5, IL-9, and IL-13 [14, 78]. These mechanisms are relevant to both human CRSwNP and asthma [17, 79, 80]. Their relevance to allergic rhinitis is also under investigation. TSLP production by human nasal epithelial cells in vitro was stimulated by a TLR2 ligand as well as by IL-1β and TNFα. Both reduced and increased expression of IL-33 gene or protein has been detected in nasal epithelium from patients with allergic rhinitis. Asthma-associated gene, ORMDL3, is involved in regulating endoplasmic reticulum (ER) stress responses and ER-mediated calcium signaling [65, 81]. In addition, Orm proteins form complexes with serine palmitoyl-CoA transferase (SPT), the first and rate-limiting enzyme in sphingolipid production, and might thereby help coordinate lipid metabolism in the secretory pathway [82]. Mucins travel from the ER to the Golgi where they are *O*-glycosylated multimerized and packed, before being secreted by exocytosis [65]. Whether epithelial ER functions and mucin production play a role in the development of chronic upper airway diseases needs to be proven.

### Intercellular Junctions

Inhaled air particles can disrupt barrier integrity, which might represent a risk factor for allergen sensitization and infections [42]. Tight junctions (TJs) consist claudins and occludins. TJs are located most apically and inhibit solute and water flow through the paracellular space, thus establishing cell polarity. They link to the actin cytoskeleton. Epithelium membrane protein 1 (EMP1) is a tight junction protein which might mediate cell proliferation [83]. The levels of EMP1 mRNA and immunostaining were lower in nasal polyp epithelium compared to control nasal mucosa. Corticosteroid treatment and mild histological findings increased the mRNA levels indicating putatively epithelial healing [83]. Soyka et al. detected a decreased *trans*-tissue resistance in biopsy specimens from patients with CRSwNP along with decreased TJ proteins occludin and zonula occluden expression by immunofluorescence, Western blotting, and RTq-PCR [84]. Adherens junctions (AJs) are located below the TJs in the lateral membrane. AJs are especially important for the maintenance of cell-cell adhesion and are comprised of the cadherin and nectin families. In epithelial cells, E-cadherin binds to intracellular catenins, thus linking AJs with the signaling system from outside to the inside of the cell. This evolutionarily conserved pathway regulates gene expression and chromatin structure implicated in epithelial wound repair responses and differentiation. Sustained loss of E-cadherin leads to epithelial-mesenchymal transition (EMT) [42].

### Self-Renewal

Nasal epithelial repair and remodeling is a highly organized process leading to necessary self-renewal after injury [85]. The normal upper airway epithelium comprises ciliated cells (representing 50–90 % of cells), mucus-secreting goblet cells, and basal cells (6–30 %) which are considered as stem cells [86•]. Both epithelial specification and terminal differentiation are critical to epithelial homeostasis. Upon injury, nondifferentiated basal cells migrate and proliferate into ciliated and goblet cells in injured regions. Moreover, epithelial cells may dedifferentiate through squamous metaplasia or epithelial to mesenchymal transition (EMT), which describes a rapid and normally reversible modulation of the epithelial phenotype toward mesenchymal cells [86•]. Epithelial cells undergoing EMT lose cell-cell polarity and adhesion to become migratory and usually downregulate junctional proteins (such as E-cadherin) while modulating their cytoskeleton organization and acquiring mesenchymal features such as alpha-smooth muscle actin (SMA), vimentin, matrix metalloproteinases (MMPs), and the transcription factors SNAIL, SLUG, and TWIST [86•, 87]. Aberrant repair leads to basal-cell and goblet-cell hyperplasia and has been found in chronic upper airway diseases [85]. De Borja Callejas et al. performed electron microscopy, immunocytochemistry, ELISA, and cytometric bead array for primary nasal epithelial cells that were cultured in an air-liquid interface. They detected that β-tubulin IV- and MUC5AC-positive cells increased and ΔNp63-positive cells decreased, indicating re-differentiation. They also found that when being fully differentiated, nasal polyp epithelium secreted more IL-8 and GMCSF than control epithelia [88]. Hupin et al. showed by immunohistochemistry and RT-qPCR a reduction of E-cadherin, high molecular weight CKs, and CK5 in CRSwNP and/or CRSsNP. This epithelial reprogramming correlates with subepithelial fibrosis and with disease severity and was not due to changes in lineage specification [86•].

The proprotein convertases (PCs) are serine proteases responsible for the proteolytic maturation of many precursor proteins involved in upper airway remodeling. PC1/3 overexpression induces in vitro morphological and phenotypic epithelial-mesenchymal transition changes of airway epithelial cells and putatively contributing to the pathogenesis of nasal polyps [87]. Host cell metabolism is manipulated by viruses for their replication and is usually followed by host cell destruction. However, early cell death, by apoptosis, can reduce virus replication and is an important factor in augmenting antigen presentation and host immune response [85]. Jeffe et al. showed in nasal tissue and cultured cells by PCR, immunohistochemistry, Western blotting, and immunofluorescence that BP180, a critical anchoring protein in keratinocytes, is widely expressed in nasal epithelium. Nonpathologic anti-BP180 antibodies were elevated in sera from CRS patients, although the authors conclude that this association may be linked to atopy [89]. Significantly higher levels of anti-dsDNA antibodies have been shown to correlate with recurrent nasal polyps requiring revision surgery [90]. Further evidence is needed to establish the role of autoimmune response in the pathogenesis of CRS.

## Conclusions and Future Needs

Sinonasal tissues are available and environmental effects can be induced in vivo, which allows performing high-quality experiments in human. There is a high need to study which epithelial mechanisms are crucial in the development of the disease and which are consequential? Systems biology approach is essential in the search for the upstream events of chronic upper airway diseases on the pathway level. An important goal in the future would be to study the biological network between environment, airway epithelium, and immune and nervous systems as a whole. In order to get a more comprehensive picture, better phenotyping and usage of endotypes of the disease would be mandatory. For instance, sensitization to multiple allergens seems to associate with a more severe phenotype in certain populations, thus atopy or allergic rhinitis seems not to be a dichotomous value, not to mention the challenges of phenotyping NAR or CRS [3, 91]. The development of functional proteomics and lipidomics, as well as that of sequencing and computational methods, will improve our ability to customize patient-specific strategies to prevent, to detect, and to treat the chronic upper airway diseases.

## References

[1] Bousquet J, Schunemann HJ, Samolinski B, Demoly P, Baena-Cagnani CE, Bachert C (2012). Allergic Rhinitis and its Impact on Asthma (ARIA): achievements in 10 years and future needs. J Allergy Clin Immunol.

[2] Fokkens WJ, Lund VJ, Mullol J, Bachert C, Alobid I, Baroody F, et al. European position paper on rhinosinusitis and nasal polyps 2012. Rhinol Suppl. 2012 Mar;(23)(23):3 p preceding table of contents, 1–298. 22764607

[3] Simpson A, Tan VY, Winn J, Svensen M, Bishop CM, Heckerman DE (2010). Beyond atopy: multiple patterns of sensitization in relation to asthma in a birth cohort study. Am J Respir Crit Care Med.

[4] Kurukulaaratchy RJ, Zhang H, Patil V, Raza A, Karmaus W, Ewart S, et al. Identifying the heterogeneity of young adult rhinitis through cluster analysis in the Isle of Wight birth cohort. J Allergy Clin Immunol. 2014;29. 10.1016/j.jaci.2014.06.017 PMC4289086 25085342

[5] Juhn YJ (2014). Risks for infection in patients with asthma (or other atopic conditions): is asthma more than a chronic airway disease?. J Allergy Clin Immunol.

[6] van Rijswijk JB, Blom HM, Fokkens WJ (2005). Idiopathic rhinitis, the ongoing quest. Allergy.

[7] Andiappan AK, Puan KJ, Lee B, Nardin A, Poidinger M, Connolly J (2014). Allergic airway diseases in a tropical urban environment are driven by dominant mono-specific sensitization against house dust mites. Allergy.

[8] Larenas-Linnemann D, Michels A, Dinger H, Shah-Hosseini K, Mosges R, Arias-Cruz A (2014). Allergen sensitization linked to climate and age, not to intermittent-persistent rhinitis in a cross-sectional cohort study in the (sub)tropics. Clin Transl Allergy.

[9] Bousquet J, Fokkens W, Burney P, Durham SR, Bachert C, Akdis CA (2008). Important research questions in allergy and related diseases: nonallergic rhinitis: a GA2LEN paper. Allergy.

[10] Scarupa MD, Kaliner MA (2009). Nonallergic rhinitis, with a focus on vasomotor rhinitis: clinical importance, differential diagnosis, and effective treatment recommendations. World Allergy Organ J.

[11] Rondon C, Campo P, Togias A, Fokkens WJ, Durham SR, Powe DG (2012). Local allergic rhinitis: concept, pathophysiology, and management. J Allergy Clin Immunol.

[12] Van Gerven L, Alpizar YA, Wouters MM, Hox V, Hauben E, Jorissen M (2014). Capsaicin treatment reduces nasal hyperreactivity and transient receptor potential cation channel subfamily V, receptor 1 (TRPV1) overexpression in patients with idiopathic rhinitis. J Allergy Clin Immunol.

[13] Hastan D, Fokkens WJ, Bachert C, Newson RB, Bislimovska J, Bockelbrink A (2011). Chronic rhinosinusitis in Europe—an underestimated disease. A GA(2)LEN study. Allergy.

[14] Scadding G (2014). Cytokine profiles in allergic rhinitis. Curr Allergy Asthma Rep.

[15] Mattila P, Renkonen J, Toppila-Salmi S, Parviainen V, Joenvaara S, Alff-Tuomala S (2010). Time-series nasal epithelial transcriptomics during natural pollen exposure in healthy subjects and allergic patients. Allergy.

[16] Poole A, Urbanek C, Eng C, Schageman J, Jacobson S, O’Connor BP (2014). Dissecting childhood asthma with nasal transcriptomics distinguishes subphenotypes of disease. J Allergy Clin Immunol.

[17] Shaw JL, Fakhri S, Citardi MJ, Porter PC, Corry DB, Kheradmand F (2013). IL-33-responsive innate lymphoid cells are an important source of IL-13 in chronic rhinosinusitis with nasal polyps. Am J Respir Crit Care Med.

[18] Trankner D, Hahne N, Sugino K, Hoon MA, Zuker C (2014). Population of sensory neurons essential for asthmatic hyperreactivity of inflamed airways. Proc Natl Acad Sci U S A.

[19] Portelli MA, Hodge E, Sayers I. Genetic risk factors for the development of allergic disease identified by genome wide association. Clin Exp Allergy. 2014 Apr 26. 10.1111/cea.12327 PMC4298800 24766371

[20] Davila I, Mullol J, Ferrer M, Bartra J, del Cuvillo A, Montoro J (2009). Genetic aspects of allergic rhinitis. J Investig Allergol Clin Immunol.

[21] Veskitkul J, Vichyanond P, Visitsunthorn N, Jirapongsananuruk O (2013). The development of allergic rhinitis in children previously diagnosed as nonallergic rhinitis. Am J Rhinol Allergy.

[22] Delagrand A, Gilbert-Dussardier B, Burg S, Allano G, Gohler-Desmonts C, Lebreton JP (2008). Nasal polyposis: is there an inheritance pattern? A single family study. Rhinology.

[23] Ramasamy A, Curjuric I, Coin LJ, Kumar A, McArdle WL, Imboden M (2011). A genome-wide meta-analysis of genetic variants associated with allergic rhinitis and grass sensitization and their interaction with birth order. J Allergy Clin Immunol.

[24] Andiappan AK, de Wang Y, Anantharaman R, Parate PN, Suri BK, Low HQ (2011). Genome-wide association study for atopy and allergic rhinitis in a Singapore Chinese population. PLoS One.

[25.•] Hinds DA, McMahon G, Kiefer AK, Do CB, Eriksson N, Evans DM (2013). A genome-wide association meta-analysis of self-reported allergy identifies shared and allergy-specific susceptibility loci. Nat Genet.

[26.•] Bunyavanich S, Schadt EE, Himes BE, Lasky-Su J, Qiu W, Lazarus R (2014). Integrated genome-wide association, coexpression network, and expression single nucleotide polymorphism analysis identifies novel pathway in allergic rhinitis. BMC Med Genomics.

[27] Bonnelykke K, Matheson MC, Pers TH, Granell R, Strachan DP, Alves AC (2013). Meta-analysis of genome-wide association studies identifies ten loci influencing allergic sensitization. Nat Genet.

[28] Besnard V, Wert SE, Kaestner KH, Whitsett JA (2005). Stage-specific regulation of respiratory epithelial cell differentiation by Foxa1. Am J Physiol Lung Cell Mol Physiol.

[29] Zhao P, Zhong W, Ying X, Yao B, Yuan Z, Fu J (2010). Comparative proteomic analysis of anti-benzo(a)pyrene-7,8-dihydrodiol-9,10-epoxide-transformed and normal human bronchial epithelial G0/G1 cells. Chem Biol Interact.

[30] Kim DY, Fukuyama S, Nagatake T, Takamura K, Kong IG, Yokota Y (2012). Implications of nasopharynx-associated lymphoid tissue (NALT) in the development of allergic responses in an allergic rhinitis mouse model. Allergy.

[31] Damera G, Xia B, Sachdev GP (2006). IL-4 induced MUC4 enhancement in respiratory epithelial cells in vitro is mediated through JAK-3 selective signaling. Respir Res.

[32] Hackett NR, Shaykhiev R, Walters MS, Wang R, Zwick RK, Ferris B (2011). The human airway epithelial basal cell transcriptome. PLoS One.

[33] Bogefors J, Rydberg C, Uddman R, Fransson M, Mansson A, Benson M (2010). Nod1, Nod2 and Nalp3 receptors, new potential targets in treatment of allergic rhinitis?. Allergy.

[34] Nilsson D, Henmyr V, Hallden C, Sall T, Kull I, Wickman M (2014). Replication of genomewide associations with allergic sensitization and allergic rhinitis. Allergy.

[35] Aguilera-Aguirre L, Bacsi A, Saavedra-Molina A, Kurosky A, Sur S, Boldogh I (2009). Mitochondrial dysfunction increases allergic airway inflammation. J Immunol.

[36] Bosse Y, Bacot F, Montpetit A, Rung J, Qu HQ, Engert JC (2009). Identification of susceptibility genes for complex diseases using pooling-based genome-wide association scans. Hum Genet.

[37] Zhang Y, Endam LM, Filali-Mouhim A, Zhao L, Desrosiers M, Han D (2012). Polymorphisms in RYBP and AOAH genes are associated with chronic rhinosinusitis in a Chinese population: a replication study. PLoS One.

[38] Tournas A, Mfuna L, Bosse Y, Filali-Mouhim A, Grenier JP, Desrosiers M (2010). A pooling-based genome-wide association study implicates the p73 gene in chronic rhinosinusitis. J Otolaryngol Head Neck Surg.

[39] Li CW, Shi L, Zhang KK, Li TY, Lin ZB, Lim MK (2011). Role of p63/p73 in epithelial remodeling and their response to steroid treatment in nasal polyposis. J Allergy Clin Immunol.

[40] Mfuna-Endam L, Zhang Y, Desrosiers MY (2011). Genetics of rhinosinusitis. Curr Allergy Asthma Rep.

[41] Golebski K, Roschmann KI, Toppila-Salmi S, Hammad H, Lambrecht BN, Renkonen R (2013). The multi-faceted role of allergen exposure to the local airway mucosa. Allergy.

[42] Georas SN, Rezaee F (2014). Epithelial barrier function: at the frontline of asthma immunology and allergic airway inflammation. J Allergy Clin Immunol.

[43.•] Post S, Nawijn MC, Jonker MR, Kliphuis N, van den Berge M, van Oosterhout AJ (2013). House dust mite-induced calcium signaling instigates epithelial barrier dysfunction and CCL20 production. Allergy.

[44] Hackett TL, de Bruin HG, Shaheen F, van den Berge M, van Oosterhout AJ, Postma DS (2013). Caveolin-1 controls airway epithelial barrier function. Implications for asthma. Am J Respir Cell Mol Biol.

[45] Clarke DL, Davis NH, Campion CL, Foster ML, Heasman SC, Lewis AR (2014). Dectin-2 sensing of house dust mite is critical for the initiation of airway inflammation. Mucosal Immunol.

[46] Joenvaara S, Mattila P, Renkonen J, Makitie A, Toppila-Salmi S, Lehtonen M (2009). Caveolar transport through nasal epithelium of birch pollen allergen Bet v 1 in allergic patients. J Allergy Clin Immunol.

[47] Roschmann KI, Luiten S, Jonker MJ, Breit TM, Fokkens WJ, Petersen A (2011). Timothy grass pollen extract-induced gene expression and signalling pathways in airway epithelial cells. Clin Exp Allergy.

[48] Wagener AH, Zwinderman AH, Luiten S, Fokkens WJ, Bel EH, Sterk PJ (2014). dsRNA-induced changes in gene expression profiles of primary nasal and bronchial epithelial cells from patients with asthma, rhinitis and controls. Respir Res.

[49.•] Golebski K, Luiten S, van Egmond D, de Groot E, Roschmann KI, Fokkens WJ (2014). High degree of overlap between responses to a virus and to the house dust mite allergen in airway epithelial cells. PLoS One.

[50] Blumenthal MN (2012). Genetic, epigenetic, and environmental factors in asthma and allergy. Ann Allergy Asthma Immunol.

[51] Raedler D, Schaub B (2014). Immune mechanisms and development of childhood asthma. Lancet Respir Med.

[52] Forno E, Celedon JC (2012). Predicting asthma exacerbations in children. Curr Opin Pulm Med.

[53] Buro-Auriemma LJ, Salit J, Hackett NR, Walters MS, Strulovici-Barel Y, Staudt MR (2013). Cigarette smoking induces small airway epithelial epigenetic changes with corresponding modulation of gene expression. Hum Mol Genet.

[54] Pritchard CC, Cheng HH, Tewari M (2012). MicroRNA profiling: approaches and considerations. Nat Rev Genet.

[55] Rager JE, Moeller BC, Doyle-Eisele M, Kracko D, Swenberg JA, Fry RC (2013). Formaldehyde and epigenetic alterations: microRNA changes in the nasal epithelium of nonhuman primates. Environ Health Perspect.

[56] McKiernan PJ, Molloy K, Cryan SA, McElvaney NG, Greene CM (2014). Long noncoding RNA are aberrantly expressed in vivo in the cystic fibrosis bronchial epithelium. Int J Biochem Cell Biol.

[57] Lee RJ, Chen B, Doghramji L, Adappa ND, Palmer JN, Kennedy DW (2013). Vasoactive intestinal peptide regulates sinonasal mucociliary clearance and synergizes with histamine in stimulating sinonasal fluid secretion. FASEB J.

[58] Choksi SP, Babu D, Lau D, Yu X, Roy S (2014). Systematic discovery of novel ciliary genes through functional genomics in the zebrafish. Development.

[59] Lee RJ, Xiong G, Kofonow JM, Chen B, Lysenko A, Jiang P (2012). T2R38 taste receptor polymorphisms underlie susceptibility to upper respiratory infection. J Clin Invest.

[60.•] Li YY, Li CW, Chao SS, Yu FG, Yu XM, Liu J, et al. Impairment of cilia architecture and ciliogenesis in hyperplastic nasal epithelium from nasal polyps. J Allergy Clin Immunol. 2014 Sep 4. *Aberrant ciliogenesis and primary motile cilia impairment were found in isolated and cultured nasal epithelial stem cells more frequently in patients with CRSwNP than in controls.* 10.1016/j.jaci.2014.07.038 25201258

[61] Haxel BR, Schafer D, Klimek L, Mann WJ (2001). Prostaglandin E2 activates the ciliary beat frequency of cultured human nasal mucosa via the second messenger cyclic adenosine monophosphate. Eur Arch Otorhinolaryngol.

[62] Hattori R, Shimizu S, Majima Y, Shimizu T (2009). Prostaglandin E2 receptor EP2, EP3, and EP4 agonists inhibit antigen-induced mucus hypersecretion in the nasal epithelium of sensitized rats. Ann Otol Rhinol Laryngol.

[63] Liu T, Laidlaw TM, Katz HR, Boyce JA (2013). Prostaglandin E2 deficiency causes a phenotype of aspirin sensitivity that depends on platelets and cysteinyl leukotrienes. Proc Natl Acad Sci U S A.

[64] Button B, Cai LH, Ehre C, Kesimer M, Hill DB, Sheehan JK (2012). A periciliary brush promotes the lung health by separating the mucus layer from airway epithelia. Science.

[65] Erle DJ, Sheppard D (2014). The cell biology of asthma. J Cell Biol.

[66] Birket SE, Chu KK, Liu L, Houser GH, Diephuis BJ, Wilsterman EJ (2014). A functional anatomic defect of the cystic fibrosis airway. Am J Respir Crit Care Med.

[67] Purkey MT, Li J, Mentch F, Grant SF, Desrosiers M, Hakonarson H (2014). Genetic variation in genes encoding airway epithelial potassium channels is associated with chronic rhinosinusitis in a pediatric population. PLoS One.

[68] Tomazic PV, Birner-Gruenberger R, Leitner A, Obrist B, Spoerk S, Lang-Loidolt D (2014). Nasal mucus proteomic changes reflect altered immune responses and epithelial permeability in patients with allergic rhinitis. J Allergy Clin Immunol.

[69] Tieu DD, Kern RC, Schleimer RP (2009). Alterations in epithelial barrier function and host defense responses in chronic rhinosinusitis. J Allergy Clin Immunol.

[70] Richer SL, Truong-Tran AQ, Conley DB, Carter R, Vermylen D, Grammer LC (2008). Epithelial genes in chronic rhinosinusitis with and without nasal polyps. Am J Rhinol.

[71] Seshadri S, Lin DC, Rosati M, Carter RG, Norton JE, Suh L (2012). Reduced expression of antimicrobial PLUNC proteins in nasal polyp tissues of patients with chronic rhinosinusitis. Allergy.

[72] Wei Y, Xia W, Ye X, Fan Y, Shi J, Wen W (2014). The antimicrobial protein short palate, lung, and nasal epithelium clone 1 (SPLUNC1) is differentially modulated in eosinophilic and noneosinophilic chronic rhinosinusitis with nasal polyps. J Allergy Clin Immunol.

[73] Hupin C, Rombaux P, Bowen H, Gould H, Lecocq M, Pilette C (2013). Downregulation of polymeric immunoglobulin receptor and secretory IgA antibodies in eosinophilic upper airway diseases. Allergy.

[74] Hammad H, Chieppa M, Perros F, Willart MA, Germain RN, Lambrecht BN (2009). House dust mite allergen induces asthma via Toll-like receptor 4 triggering of airway structural cells. Nat Med.

[75] Fransson M, Adner M, Erjefalt J, Jansson L, Uddman R, Cardell LO (2005). Up-regulation of Toll-like receptors 2, 3 and 4 in allergic rhinitis. Respir Res.

[76.•] Tengroth L, Millrud CR, Kvarnhammar AM, Kumlien Georen S, Latif L, Cardell LO (2014). Functional effects of Toll-like receptor (TLR)3, 7, 9, RIG-I and MDA-5 stimulation in nasal epithelial cells. PLoS One.

[77] Tengroth L, Arebro J, Kumlien Georen S, Winqvist O, Cardell LO (2014). Deprived TLR9 expression in apparently healthy nasal mucosa might trigger polyp-growth in chronic rhinosinusitis patients. PLoS One.

[78] Licona-Limon P, Kim LK, Palm NW, Flavell RA (2013). TH2, allergy and group 2 innate lymphoid cells. Nat Immunol.

[79] Saglani S, Lui S, Ullmann N, Campbell GA, Sherburn RT, Mathie SA (2013). IL-33 promotes airway remodeling in pediatric patients with severe steroid-resistant asthma. J Allergy Clin Immunol.

[80] Shikotra A, Choy DF, Ohri CM, Doran E, Butler C, Hargadon B (2012). Increased expression of immunoreactive thymic stromal lymphopoietin in patients with severe asthma. J Allergy Clin Immunol.

[81] Cantero-Recasens G, Fandos C, Rubio-Moscardo F, Valverde MA, Vicente R (2010). The asthma-associated ORMDL3 gene product regulates endoplasmic reticulum-mediated calcium signaling and cellular stress. Hum Mol Genet.

[82] Breslow DK, Collins SR, Bodenmiller B, Aebersold R, Simons K, Shevchenko A (2010). Orm family proteins mediate sphingolipid homeostasis. Nature.

[83] Yu XM, Li CW, Li YY, Liu J, Lin ZB, Li TY (2013). Down-regulation of EMP1 is associated with epithelial hyperplasia and metaplasia in nasal polyps. Histopathology.

[84] Soyka MB, Wawrzyniak P, Eiwegger T, Holzmann D, Treis A, Wanke K (2012). Defective epithelial barrier in chronic rhinosinusitis: the regulation of tight junctions by IFN-gamma and IL-4. J Allergy Clin Immunol.

[85] Yan Y, Gordon WM, Wang DY (2013). Nasal epithelial repair and remodeling in physical injury, infection, and inflammatory diseases. Curr Opin Otolaryngol Head Neck Surg.

[86.•] Hupin C, Gohy S, Bouzin C, Lecocq M, Polette M, Pilette C. Features of mesenchymal transition in the airway epithelium from chronic rhinosinusitis. Allergy. 2014 Aug 7. *Features of epithelial dedifferentiation towards a mesenchymal phenotype are observed in CRSwNP and CRSsNP and correlate with airway fibrosis and inflammation.* 10.1111/all.12503 25104359

[87] Lee SN, Lee DH, Sohn MH, Yoon JH (2013). Overexpressed proprotein convertase 1/3 induces an epithelial-mesenchymal transition in airway epithelium. Eur Respir J.

[88] de Borja CF, Martinez-Anton A, Alobid I, Fuentes M, Cortijo J, Picado C (2014). Reconstituted human upper airway epithelium as 3-d in vitro model for nasal polyposis. PLoS One.

[89] Jeffe JS, Seshadri S, Hamill KJ, Huang JH, Carter R, Suh L (2013). A role for anti-BP180 autoantibodies in chronic rhinosinusitis. Laryngoscope.

[90] Tan BK, Li QZ, Suh L, Kato A, Conley DB, Chandra RK (2011). Evidence for intranasal antinuclear autoantibodies in patients with chronic rhinosinusitis with nasal polyps. J Allergy Clin Immunol.

[91] Just J, Saint-Pierre P, Gouvis-Echraghi R, Laoudi Y, Roufai L, Momas I (2014). Childhood allergic asthma is not a single phenotype. J Pediatr.

